# Evaluation of the Composition, Thermal and Mechanical Behavior, and Color Changes of Artificially and Naturally Aged Polymers for the Conservation of Stained Glass Windows

**DOI:** 10.3390/polym15122595

**Published:** 2023-06-07

**Authors:** Josef Brandt, Elisavet Kanaki, Dieter Fischer, Christoph Herm

**Affiliations:** 1 Center Macromolecular Structure Analysis, Institute of Macromolecular Chemistry, Leibniz Institute of Polymer Research Dresden, 01069 Dresden, Germany; josefbrandt@posteo.de (J.B.); kanaki@ipfdd.de (E.K.); fisch@ipfdd.de (D.F.); 2 Study Program of Restoration, Dresden University of Fine Arts, 01307 Dresden, Germany

**Keywords:** stained glass, conservation, Naumburg Cathedral, acrylate, lamination, epoxy, FTIR, Raman, glass transition temperature, artificial aging, thermal analysis, adhesive strength

## Abstract

Investigations of historical conservation materials on historical stained glass windows of the Naumburg Cathedral in Germany offered an opportunity for the study of polymers, naturally aged in a non-controlled environment. This allowed the conservation history of the cathedral to be traced and expanded by valuable insights. The historical materials were characterized through the use of spectroscopy (FTIR, Raman), thermal analysis, PY-GC/MS, and SEC on taken samples. The analyses show that acrylate resins were predominantly used for conservation. The lamination material from the 1940s is particularly noteworthy. Epoxy resins were also identified in isolated cases. Artificial aging was used to investigate the influence of environmental influences on the properties of the identified materials. Through a multi-stage aging program, influences of UV radiation, high temperatures and high humidity can be considered in isolation. Piaflex F20, Epilox, Paraloid B72 as a modern material and combinations of Paraloid B72/diisobutyl phthalate and PMA/diisobutyl phthalate were investigated. The parameters yellowing, FTIR spectra, Raman spectra, molecular mass and conformation, glass transition temperature, thermal behavior, and adhesive strength on glass were determined. The effects of the environmental parameters on the investigated materials are differentiated. UV and extreme temperatures tend to show a stronger influence than humidity. The comparison of the artificially aged samples with the naturally aged samples from the cathedral shows that the latter were less aged. Recommendations for the conservation of the historical stained glass windows were derived from the results of the investigation.

## 1. Introduction

Since the development of the first acrylic resins at the dawn of the 20th century, a variety of synthetic polymers has been developed. By the 1950s synthetic polymers were also dominating conservation practices in terms of cultural heritage [1,2]. Materials used in conservation applications should ideally maintain reversibility and have properties such as a suitable color, refraction index and glass transition temperature in a time scale considerably longer than that strived for in industrial applications [3]. Therefore, the study of synthetic polymers in heritage science largely focuses on aging behavior, usually relying on artificial weathering studies for the estimation of their properties over time [4,5,6,7]. In past decades, attention started to be focused on past conservation interventions for the assessment of the long-term performance of polymers under real conditions [8,9,10,11]. Although some information on past interventions can be retrieved from archives, documentation is not always adequate or reliable. A physicochemical characterization of the materials is thus indispensable [12,13,14,15]. Recently inscribed in the UNESCO World Heritage List, the 13th century Naumburg Cathedral in Saxony-Anhalt, Germany, offers an excellent opportunity for the study of historical polymers, naturally aged in a non-controlled environment. It possesses complete stained glass cycles of high artistic value in its east and west choirs. In contrast to their immeasurable value is the poor condition of the glass panels, which made carefully planned restoration or conservation measures urgently necessary. A preliminary investigation in 2014 showed that polymeric restoration materials had been introduced in earlier conservation measures during World War II and in the 1960s in the German Democratic Republic [16]. The stained glass windows of the monument have been subjected to conservation campaigns throughout the 20th century [17] that reflect the advances in conservation and in polymer science, including polymers produced and applied in the German Democratic Republic. In 1939 Richard Jacobi supervised the first documented application of an acrylic resin on stained glass [18], a predecessor of the lamination process later developed at the Cologne Cathedral and known as “Doublierung” [19]. The process consisted of consolidation of grissaille painting followed by protection of the glass surface with a supporting glass, using polyacrylic acid esters in both steps. In the course of this campaign, the window panels were disassembled and stored in cellars for protection during World War II. They remained in storage until the 1960s when they were restored during a large-scale campaign involving consolidation, bonding and reassembling of the partly severely damaged glass. Less extensive conservation interventions have followed since.

**Figure 1:**
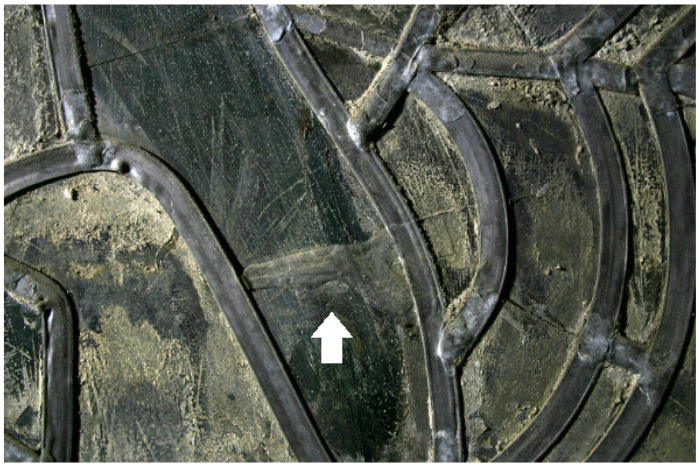
Stained glass window in Naumbug Cathedral: acrylate bond (center, analyzed as Piaflex F20) (arrow).

**Figure 2:**
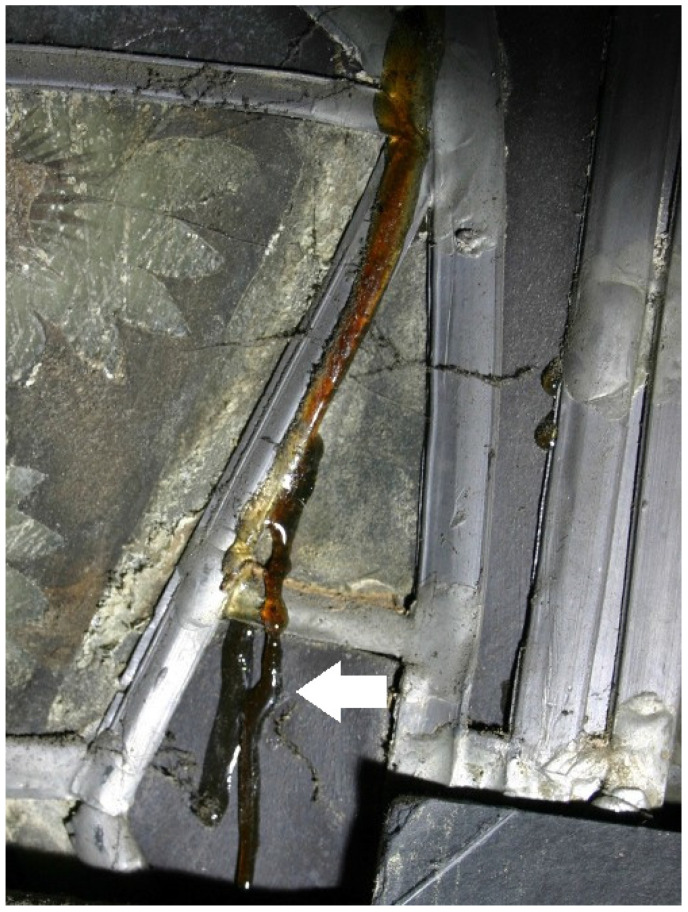
Stained glass window in Naumburg Cathedral: brown-yellow laminating polymer (analyzed as PMA/DiBP), leaking between glass sheets (arrow).

**Figure 3:**
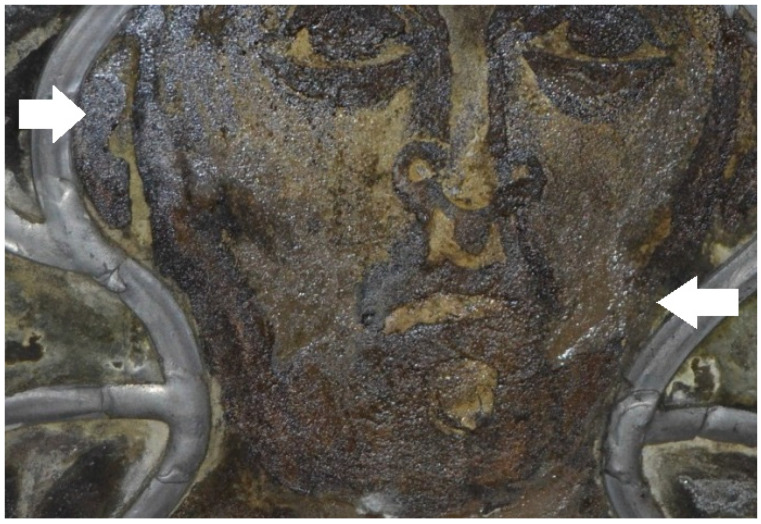
Stained glass window in Naumburg Cathedral: Interior surface with grisaille, consolidated with polymer (analyzed as PMA) (arrows).

**Figure 4:**
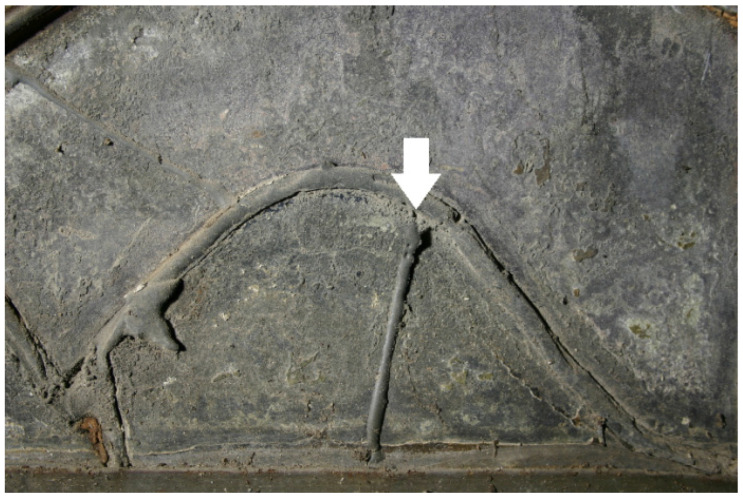
Stained glass window in Naumburg Cathedral: opaque epoxy bond (arrow).

Parallel to the sampling in November 2016, the effect of the restoration measures on the object was documented [16]. The acrylics used to secure cracked glass are, in many cases, deformed and no longer adhere to the substrate (Figure 1). If the glass substrate has a loose or very corroded surface, its damage cannot be ruled out. On existing laminations, it has been observed that the polymer flows down along the glass sheets (Figure 2). On the other hand, the consolidations of surfaces with acrylates are still in good condition (Figure 3). The bonds with epoxy resin are still stable (Figure 4).

To facilitate the approach to historical conservation materials in future conservation work, a scientific research program was established. The aim of this study was to facilitate a decision on whether the preservatives already in use still serve their purpose after a long service life or need to be replaced. Based on the analytical findings on samples taken from the historical materials, polymer-glass composite specimens of representative materials were prepared and artificially aged. Artificial aging allows for the estimation of the aging state of the polymers found in the cathedral. These investigations into the materials historically used in Naumburg Cathedral are novel and of great practical importance, as these materials were applied in numerous objects in the former German Democratic Republic and neighboring countries during the second half of the 20th century.

The evaluation of protective agents, including polymers, by artificial aging has also been established in preservation science for decades [3,9]. In a long-term study to evaluate the effect of modifiers on vinylacetate–ethylene copolymers, Down [20] combined light aging with fluorescent lamps (700–800 lux and 190 µW/lm UV-A) for 8 years at room conditions (c. 22 °C/c. 45% RH) and subsequently in the dark for another 9 years. The cumulative exposure of 49–56 Mluxh corresponded to about 80 years of museum storage (at 200 lux). The photo-oxidative stability of a series of commercial acrylic/methacrylic protective resins (Paraloid-B66, -B67, -B72 and -B82) was investigated by Chiantore/Lazzari [21]. A Xenon light source with 765 W/m^2^ at wavelengths above 295 nm was applied for up to 2500 h. The maximum temperature on the samples was 45 °C. Hedlund/Johansson [22] applied aging cycles up to 1500 h, which were combined of water mist, elevated temperature and humidity (50 °C/100% RH) and irradiation with UV-A (320–380 nm) at elevated temperature (60 °C) to evaluate different polymer dispersions. The cumulative UV irradiation was equivalent to 141/282/422 kJ/m^2^. To evaluate historical coating materials for glass, Torge [23] first applied a 4-week exposure to temperature and humidity cycles (40 °C/50% RH or 10 °C/80% RH) and sulfur dioxide. After that, no damage to the coatings was observed. Subsequently, UV was applied for 885 h at wavelengths of 290–450 nm with approx. 45 W/m^2^ at 50 °C/10% RH, and the first exposure situation was repeated. To evaluate modern preservatives for glass, Marschner et al. [24] applied a single chemical treatment with a mixture of sulfuric acid and nitric acid followed by climatic cycles without irradiation under room temperature at 100% RH, 40 °C at 32% RH, and room temperature at 58% RH, repeated as needed. The application of elevated temperature to accelerate aging reactions in artificial aging creates the dilemma that reactions with higher activation energies also come into effect, which do not occur under natural conditions and thus falsify the result [25]. In the aging experiments in the literature [20,21,22,23,24], temperatures reach up to 60 °C. Alternations between low and high temperatures would facilitate additional mechanical stress and possibly the formation of micro cracks due to thermal expansion. Increased humidity or even liquid water [22] enable hydrolysis reactions, which play a major role in the aging of polymers and were absolutely to be included in the artificial aging. Although condensation on the glass panes inside the Naumburg Cathedral cannot be ruled out, the use of liquid water was dispensed with for technical reasons. Photochemical reactions are undoubtedly crucial for the aging of polymers, especially on glass windows. Thus, light and UV radiation were to be applied—as in all cited literature methods. However, irradiation through window glass was to be filtered (wavelengths above 300 nm) since the polymers in the Naumburg Cathedral were mainly applied to the inside of the glass panes. In the cathedral, elevated concentrations of sulfur dioxide, also in the interior, can be assumed in the past (until about 1990) due to the proximity of the Central German lignite firing and chemical industry. An effect on the polymers in the form of addition reactions and catalysis of hydrolysis seems fundamentally likely. However, exposure during artificial aging of the samples with gaseous sulfur dioxide is technically complex and apparently has no immediate effects [23]. Treatment with liquid acids, according to [24], appeared too harsh. Therefore, in the present study, the use of chemical stress was omitted.

## 2. Materials and Methods

### 2.1. Material Analysis

Fourier-transform infrared spectroscopy (FTIR): A Tensor-27 FTIR spectrometer coupled to a Hyperion 2000 microscope (Bruker Optics, Ettlingen, Germany) was used. Samples were analyzed in transmission in a diamond crystal. Spectra were acquired in the range of 4000–580 cm^−1^ with a resolution of 4 cm^−1^ and 32 scans per spectrum. Subsequent baseline correction was performed using the “Concave Rubberband Correction” algorithm (64 points, 10 iterations) of the OPUS 5 program. For better readability of the spectra, the CO_2_ bands in the range 2390–2285 cm^−1^ have been removed in the following diagrams.

Raman spectroscopy (RS): The confocal Raman microscope alpha300R (WITEC, Ulm, Germany) was used. The excitation wavelength of the laser used was selected specific to the sample. For stronger fluorescence appearances, measurements were made at 785 nm, otherwise at 532 nm, with a spectral resolution of 2 cm^−1^.

Pyrolysis-GC/MS (Py-GC/MS): The samples were pyrolyzed using a Pyroprobe 5000 (CDS Analytical Inc., Oxford, PA, USA) at temperatures ranging from 280 to 620 °C and times of 10 or 15 s, depending on the sample) and analyzed using a GC Systems 7890 A (Agilent Technologies Inc., Santa Clara, CA, USA). The inlet temperature was 280 °C, and the column used was a nonpolar HP-5MS (30 m × 0.25 mm inner diameter × 0.25 µm film thickness) with 1 mL/min helium as the carrier gas. The GC oven temperature was maintained at 50 °C for 2 min and then heated to 280 °C at 12 °C/min. The split to the mass detector (MSD 5975C inert XL EI/CI) was 1:20, ionization was performed as EI at 70 eV, and the scan range was set to 15–550 m/z.

Size exclusion chromatography (SEC) can be used to determine the molar mass distribution. For this purpose, a modular set-up consisting of a degasser and HPLC pump (Agilent Technologies, Inc, Santa Clara, CA, USA), separation column (PLgel Mixed-C, Agilent Technologies), refractive index and viscosity detector (WGE Dr Bures, Dallgow-Döberitz, Germany) and 3-angle light scattering detector (miniDAWN, WYATT Technologies Corp., Santa Barbara, CA, USA) was used. Measurements were performed in tetrahydrofuran (THF) at room temperature and a flow rate of 1 mL/min. With the aid of the static light scattering detection used, the radius of gyration (Rg) can also be determined at each elution time. From the plot of lg Rg versus lg Mn, a conformation plot is obtained representing the scaling of the molecular size with the molecular mass average, Mn. A rigid rod would have a slope of 1, and a perfect sphere, a slope of 0.33. Typical values for linear polymers in good solvents are around 0.5–0.6. Therefore, the lower the slope of the plot, the more compact the structure of a polymer molecule, indicating a stronger degree of branching.

Thermal analysis: The thermal stability of adhesive materials is of great practical relevance since bonded joints or applied bond coats should not lose their dimensional stability at elevated temperatures. DSC measurements are suitable here for recording the glass transition temperature Tg. Differential scanning calorimetry (DSC) was performed with a DSCQ2000 (TA Instruments, New Castle, DE, USA). Two heating/cooling cycles with a heating/cooling rate of 10 °C/min were performed to determine the Tg. In the first heating cycle, heating was performed from −80 °C to 150 °C, and in the second, from −80 to 250 °C.

Thermogravimetric analysis (TGA) shows, on the one hand, at which temperatures the polymer actually degrades and, on the other hand, how much low molecular weight constituents evaporate at temperatures below the degradation temperature. TGA measurements were performed using a TGAQ5000 (TA Instruments, New Castle, DE, USA). Under a nitrogen atmosphere, the samples were heated from room temperature to 800 °C at 10 °C/min.

Colorimetric measurements were used to characterize the change in the color impression of the sample materials during aging. These were performed using a CM-3600d spectrophotometer (Konica Minolta Sensing, Nieuwegein, The Netherlands), with the diameter of the measurement spot of approximately 10 mm. The Yellowness Index (YI) was calculated according to DIN 6167 [26]. Six sample glasses each were measured in the transmission of five individual acquisitions. A final value was obtained by averaging the obtained results.

Mechanical testing: An important quality characteristic of the preservation materials investigated is their film adhesion (maximum tensile strength) to glass. To characterize the film adhesion of the films applied to the surface, pull-off tests were carried out with a PosiTest AT-A (DeFelsko, Ogdensburg, NY, USA) using 20 mm stainless steel pull-off knobs. The pull-off speed was 0.2 mm/s. The knobs were bonded with Loctite EA 9466 two-component adhesive and cured for at least three days. Raman spectroscopy on torn films on the knobs confirmed that the polymer films were thick enough to prevent diffusion of the adhesive to the glass interface. At least six individual tests were performed per sample to determine the values. Only values in which more than half of the polymer film was torn off were included in the evaluation.

In addition to the layer adhesion, the tensile strength (maximum tensile stress) of crack adhesives is of high relevance for application in glass preservation. The chosen specimen design was intended to allow testing of a defined adhesive seam with a width of 1 mm. In practice, however, the set-up reached its limits because some of the bonds were so brittle and fragile that the test specimens broke before or during clamping in the tractor. In the case of softer materials (plasticized acrylic resins), the elevated temperatures during aging caused some of the material to run out of the bonds so that the amount of polymer in the bond changed. Consistent material tests thus could not be performed with the artificially aged specimens.

### 2.2. Artificial Aging

#### 2.2.1. Model Materials

Based on the analysis results of the specimens from the Naumburg Cathedral, three representative materials were initially selected to be subjected to artificial aging on model specimens. When the lamination material was identified more precisely after the start of the first aging series, additional materials were added for the second aging series. This had two objectives:

1. A deeper understanding of the parameters influencing the aging of the polymer materials used on the Naumburg Cathedral (sunlight, UV content, and changes in temperature and humidity) and their associated changes in properties.
2. On the basis of the knowledge gained, the condition of the samples taken in the cathedral is to be assessed in a more profound way because the corresponding measurements cannot be carried out on the original material for the most part. It is particularly important for future restoration work to assess whether a material is already on the verge of failure or is likely to be able to withstand the current conditions for a certain time.

The following materials were selected for the first aging series: original Piaflex F20 (VEB Stickstoffwerk, Piesteritz, German Democratic Republic) and Epilox EGK19 (VEB Leuna-Werke “Walter Ulbricht”, Leuna, German Democratic Republic) from archived stocks; the lamination material was replicated: Paraloid B72 + 13 wt% diisobutyl phthalate (DiBP) as a plasticizer. In the second aging series, PMA + 14 wt% DiBP was added as a reference for the lamination material and pure Paraloid B72 (Rohm and Haas Deutschland, Frankfurt/Main, Germany). Poly(methylacrylate) (PMA) was synthesized radically using AIBN as an initiator in a 1,4-Dioxane solution, heated at 50 °C for 4 h. The substrate was RESTOVER glass (Schott, Mainz, Germany) in a format adapted to the aging chamber (8 cm × 14 cm).

**Figure 5:**
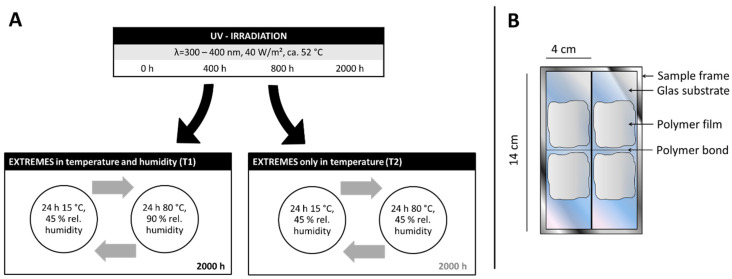
(**A**) Overview of the artificial aging procedure and (**B**) schematic representation of the used sample layout.

Each specimen holder contains two glass specimens, each measuring 4 × 14 cm^2^, which were scored in the middle using a glass cutter and then broken through (Figure 5B). Using the respective model polymers, the two halves of the model glass were rejoined with a 1 mm thick adhesive seam. In addition to the simulated crack bonding, a two-dimensional coating of the model polymers was applied to each half of the glass substrate by brushing. This resulted in films with a thickness of about 150–200 µm. Thus, the two-dimensional coatings were significantly thicker than those found in the cathedral. However, this made it possible to determine the adhesive strength of the films.

#### 2.2.2. Aging Conditions

The selection of the climatic conditions for the artificial aging was aimed at separately exposing and analyzing UV radiation, temperature, and humidity (Figure 5A). To stress the materials to failure within a practically feasible period (2000 h = approx. 2.75 months), conditions were deliberately selected thar do not actually occur in the cathedral. On the other hand, a temperature of 80 °C was chosen, which still excludes aging reactions with significantly increased activation energies [3]. The artificial weathering experiments were set up to study the effects of UV irradiation and extreme changes in temperature and humidity separately. Knowledge about which climate component affects what material property to a certain extent is of high value when conservation measures are about to be planned.

Therefore, in the first step, UV irradiation of the samples was carried out using a Ci5000 Weather-O-meter (ATLAS, Mount Prospect, IL, USA) equipped with a UV filter that cuts of hard UV light at wavelengths below 300 nm. The black panel temperature was at approx. 58 °C and the chamber temperature was approx. 52 °C throughout the entire irradiation experiment. Samples were taken after 0, 400, 800, and 2000 h and subjected to one of two subsequently performed thermal weathering programs. In T1, the temperature and humidity conditions altered between 15 °C and 45% rel. humidity and 80 °C and 90% rel. humidity in 24 h cycles for 2000 h in total. In contrast, in T2, only the temperature was altered between 15 and 80 °C whilst keeping the relative humidity at a constant level of 45% (Figure 5A).

## 3. Results and Discussion

### 3.1. Naturally Aged Historical Polymers

**Figure 6:**
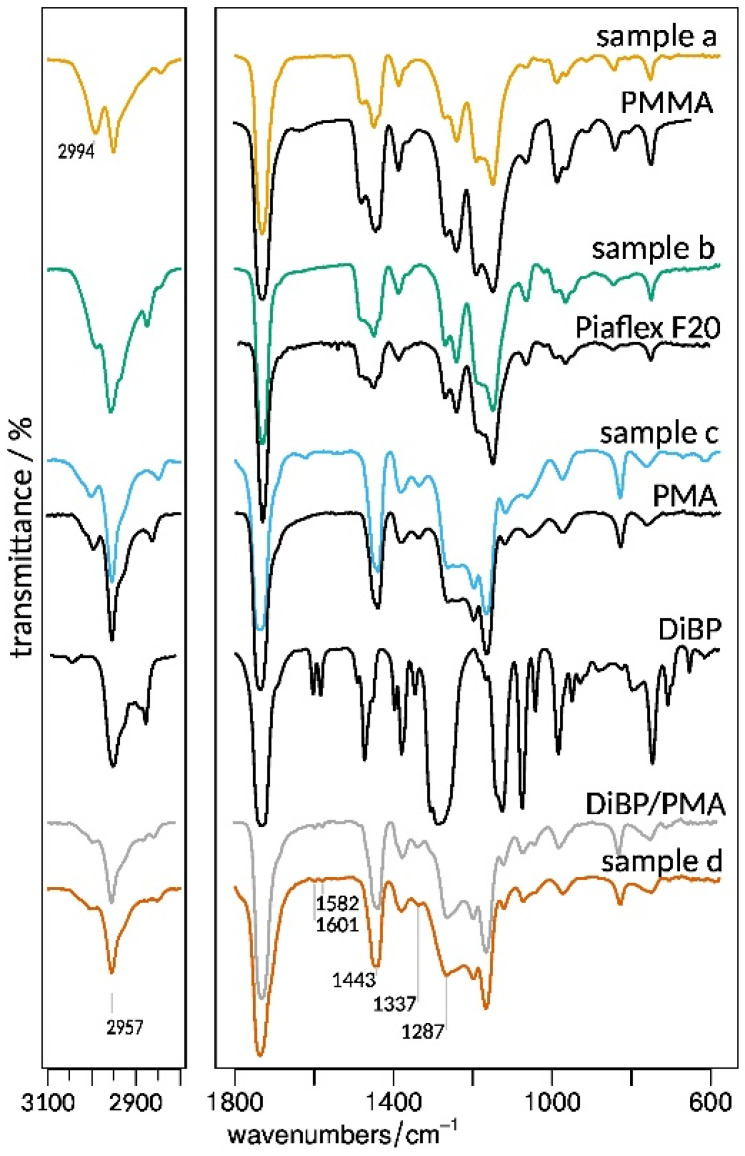
Representative FTIR transmission spectra of samples identified as acrylic and methacrylic polymers. The materials exhibit characteristic differences in the fingerprint as well as in the aliphatic C-H stretching region. (a) is in excellent agreement with PMMA, (b) Piaflex F20, (c) PMA, and (d) lamination material.

A total of 130 samples were collected from the windows of the Naumburg Cathedral. The collected material per sample ranges from a few micrograms to milligrams (for detailed information on the sampling campaign, please see Introduction). The samples were screened by means of FTIR microscopy, 64 of which contained materials related to conservation: Acrylic polymers were detected in 41 samples, epoxy resins in 5, protein glue in 12 and cellulose tissue in 19 samples. The remaining samples were dominated by glass corrosion products, mainly gypsum. Acrylics were originally used on the stained glass windows to join the fragments of broken glass for the consolidation of surfaces and as lamination material to secure the heavily fragmented original pieces on thinner supporting glass. Four spectroscopically different resins were identified on the stained glass windows. They are presented in the example of four representative samples in Figure 6.

FTIR spectra of poly(meth)acrylates are generally composed of the asymmetric stretching of aliphatic C-H in the region of 2800–3000 cm^−1^, the ester C=O stretching around 1730 cm^−1^ and a fingerprint region which is the result of various overlapping bands. The two doublet bands in the region of 1280–1150 cm^−1^ (ν(C-O-C)) in the spectra of samples (a) and (b) are characteristic of polymethacrylic polymers [27]. By contrast, the spectra of samples (c) and (d) exhibit—instead of the doublet band at 1280 cm^−1^—a plateau and a broad peak at 1287 cm^−1^, respectively. In addition, weak peaks at 1582 cm^−1^ and 1601 cm^−1^ in spectrum (d) are indicative of aromatic components’ ring stretching.

For the further reliable identification of the materials, FTIR libraries and other references were consulted. Sample (b) is in excellent agreement with Piaflex F20, a resin that was produced in the German Democratic Republic [28]. The product is described in datasheets [28] as a butyl-methacrylate-based resin; however, the measured FTIR spectrum indicates a poly-(methyl-co-butyl methacrylate)—similar to Paraloid B66 (Rohm and Haas)—rather than pure PBMA [21,29]. The spectrum of sample (a) matches poly(methyl methacrylate) (PMMA) from the IRUG database [30]. The identification of sample (d) was not as straightforward since no match was found in the databases. After poly(methyl acrylate) (PMA) was found to have a similar spectrum in the Atlas of Polymer and Plastics Analysis [31], a reference of PMA was synthesized, the spectrum of which confirmed the composition of the sample. Subsequently, sample (d) could be identified as a mixture of PMA and the plasticizer diisobutyl phthalate (DiBP) (Figure 6).

**Figure 7:**
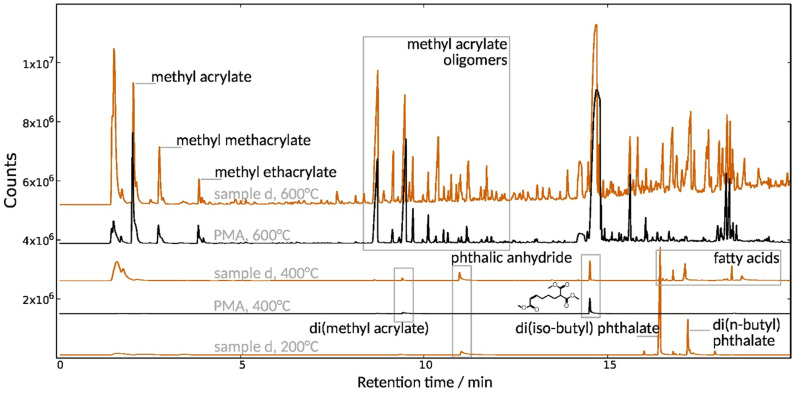
Py-GC/MS of poly(methyl acrylate) reference material and sampled lamination material (sample (d), DiBP/PMA mixture) at 200 °C, 400 °C, and 600 °C.

Py-GC-MS was performed on selected cases for direct structural analysis of the sampled polymers. An additional separation was achieved in the case of mixtures by pyrolyzing the samples at gradually increasing temperatures. This approach was particularly useful in the case of the PMA/DiBP sample (d), which was pyrolyzed in three steps (Figure 7): Already at 200 °C, the plasticizer evaporates and produces the fragments diisobutyl phthalate, dibutyl phthalate and phthalic anhydride. PMA starts fragmenting into oligomers at 400 °C; at 600 °C, it decomposes additionally into methyl acrylate monomers, while rearrangement reactions result in methyl methacrylate and ethyl acrylate fragments. The same previously reported fragmentation pattern is observed also for the PMA reference [32,33]. Both sample (b) and the Piaflex F20 reference decompose via an unzipping mechanism into their constituent monomers: methyl methacrylate and butyl methacrylate. The chromatograms are solely composed of these two peaks, thus confirming the structure as a BMA/MMA copolymer.

**Figure 8:**
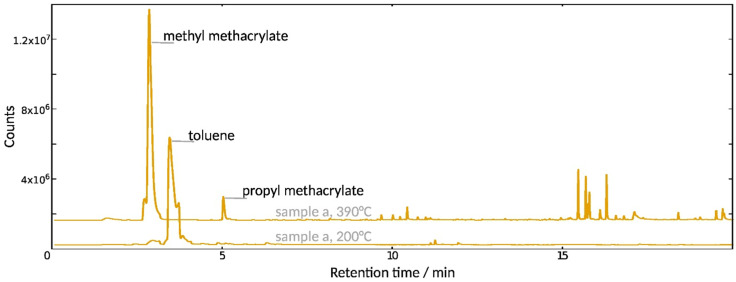
Py-GC-MS of sample (a) at 200 and 390 °C.

The approach of gradually increasing the pyrolysis temperature was also applied on sample (a), which in FTIR, matches PMMA. In this case, the volatile component separated at 200 °C is toluene, the remaining solvent used to apply the polymer at least twenty years earlier (Figure 8). Subsequent pyrolysis of the sample at 390 °C results in the fragmentation of the polymer itself into propyl methacrylate and methyl methacrylate.

Methacrylate copolymers usually depolymerize upon pyrolysis into their constituent monomers [34], as observed in sample (b). The pyrolysis fragmentation pattern of sample (a) thus suggests a poly(methyl-co-propyl methacrylate), an assumption that contradicts the FTIR result. Indeed, PMMA reportedly produces almost exclusively the methyl methacrylate monomer in a one-step unzipping reaction [35,36,37]. However, thermal degradation and accelerated UV-aging introduce double bonds and radicals in the PMMA chain [38,39], the effect of which on the fragmentation pattern has not been investigated. Since the sampled material has remained on the windows of the Cathedral for at least twenty years, its pyrolysis pathway might deviate from the reported results leading to propyl methacrylate as a secondary pyrolysis product. In the absence of information on copolymers of propyl methacrylate and on the pyrolysis mechanism of aged PMMA in the literature, the identification of the material is inconclusive.

**Figure 9:**
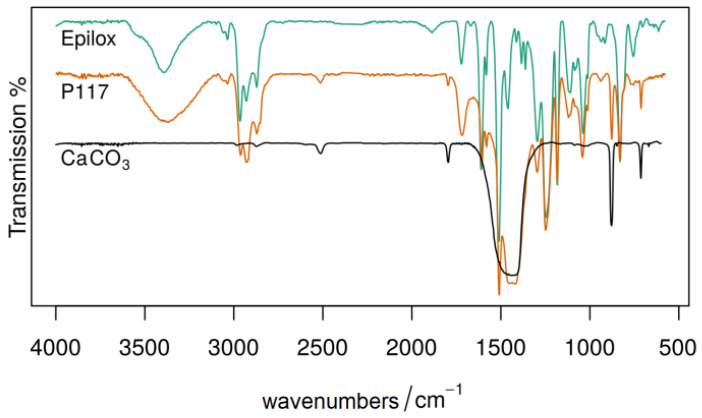
FTIR spectra of an opaque epoxy bond in transmission from sample P117 (in yellow) and references. A mixture of Epilox (in green) and calcium carbonate (in black) was identified in the FTIR spectrum.

Epoxy resin was detected exclusively on glass from the east choir. The material, which occurs at several spots, was identified spectroscopically as the commercial product Epilox on the basis of historical reference materials (Figure 9). Two of the samples from the cathedral have filler added, namely calcium carbonate or titanium dioxide, and have an optical appearance very similar to window putty.

### 3.2. Results after Artificial Aging and Discussion

#### 3.2.1. Colorimetry

**Figure 10:**
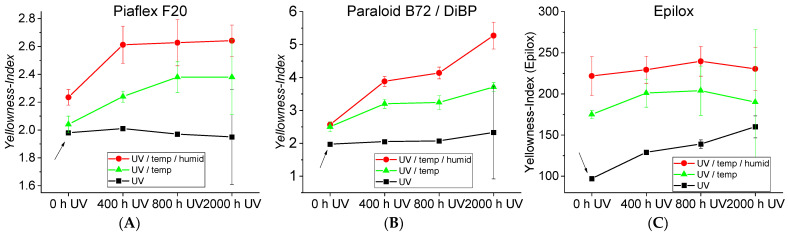
Yellowness indices of (**A**) Piaflex F20, (**B**) Paraloid B72/DiBP and (**C**) Epilox. The arrows show the respective initial state. (Note the very high initial value for Epilox).

The acrylic resins (Piaflex F20, ParaloidB72/DIBP) start with a yellowness index (YI) of about 2 before artificial aging and are thus optically classified as colorless. For both acrylics, pure UV aging has practically no effect on yellowing (Figure 10A,B). Pure temperature aging hardly leads to yellowing; somewhat stronger for the Paraloid B72/DiBP (increase to YI = c. 2.5). Additional humidity has no effect on yellowing. Aging by elevated temperature after UV aging, on the other hand, results in significant increases in yellowing, with Paraloid B72/DiBP showing considerably more than Piaflex F20 (Figure 10B). Aging due to both elevated temperature and moisture after UV aging shows an even stronger effect for both acrylics. It should be emphasized that for all acrylics, the longer the previous UV aging, the more severe the yellowing. This shows that UV aging per se does not lead to yellowing but apparently triggers inducing processes, which lead to correspondingly stronger yellowing at elevated temperature or humidity.

Unaged Epilox already shows an extremely high value of YI = 100, which is probably due to the aged state of the resin components used. It cannot be assumed that a freshly produced Epilox of the same compound would have been comparably yellow 50 years ago. In the case of Epilox, even simple UV aging results in moderate yellowing, increasing with irradiation time (YI ≤ 162) (Figure 10C). Simple aging with temperature significantly increases the yellowing. Additional humidity has another strong influence (YI ≤ approx. 225). Previous UV aging has—in contrast to the acrylics—only a slight additional influence on the yellowing, which is even slightly lower again after 2000 h UV. However, after the thermal aging programs, the color of Epilox goes strongly into the red to dark brown range, so that here the specification of a YI is no longer representative of the condition of the sample.

**Figure 11:**
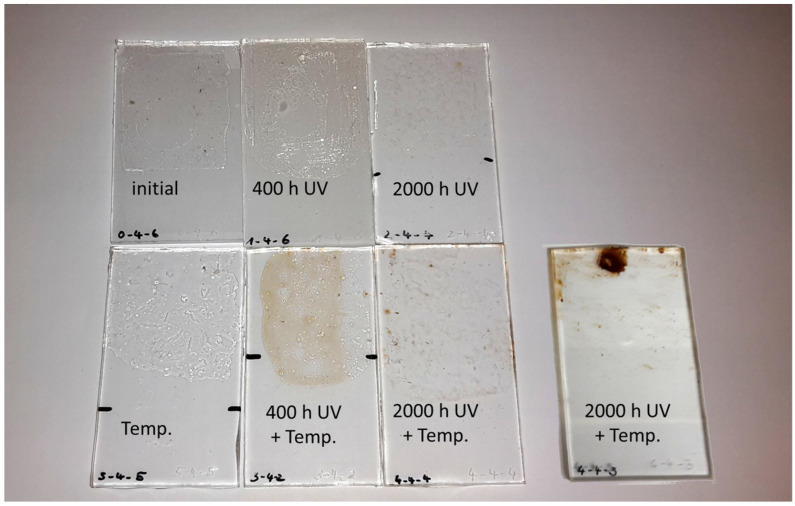
Color changes of PMA/DiBP films after different stages of artificial aging. The films started to disintegrate, rendering an accurate YI determination impossible.

In the case of the PMA/DiBP, no consistent color values could be determined since the applied film had disintegrated into smaller fragments after 2000 h of UV aging. However, a very strong yellowing of the PMA/DiBP films could be visually recognized (Figure 11), which corresponds to the appearance of the naturally aged lamination adhesive (Figure 2).

#### 3.2.2. Composition and Molecular Properties

Raman spectroscopy allows for the detection of the contained toluene and its disappearance from the polymer Piaflex F20 through the different artificial aging steps (Figure S1) while the actual polymer bands remain unchanged. The polymer itself thus seems chemically stable, although no conclusions can be drawn from the spectra about the change in end groups or branch points at low concentrations. Similarly, Raman spectroscopy of Paraloid B72/DiBP reveals essentially the disappearance of the DiBP bands, whereas the polymer bands remain unchanged. Only the appearance of a small band at 944 cm^−1^ after the combined thermal ages can be observed (Figure S2). The comparison with the spectra of Paraloid B72 without DiBP shows a similar picture: the polymer bands remain unchanged while bands of the solvent toluene decrease. The formation of a band at 944 cm^−1^ is not visible (Figure S4).

Raman spectroscopy on Epilox after UV aging (Figure S3) shows little change in the polymer bands and a slight decrease in the band of free epoxy groups at 915 cm^−1^, indicating post-crosslinking of the epoxy. The decrease at 915 cm^−1^ is significant in the FTIR spectra (Figures S5 and S6), which in addition shows that post-crosslinking is equally induced by thermal aging. Post-crosslinking has already been considered as a cause for the increase in Tg and is thus confirmed experimentally. The post-crosslinking is probably due to the elevated temperatures (temperature during UV aging is about 52 °C) and the associated increased chain mobility.

**Figure 12:**
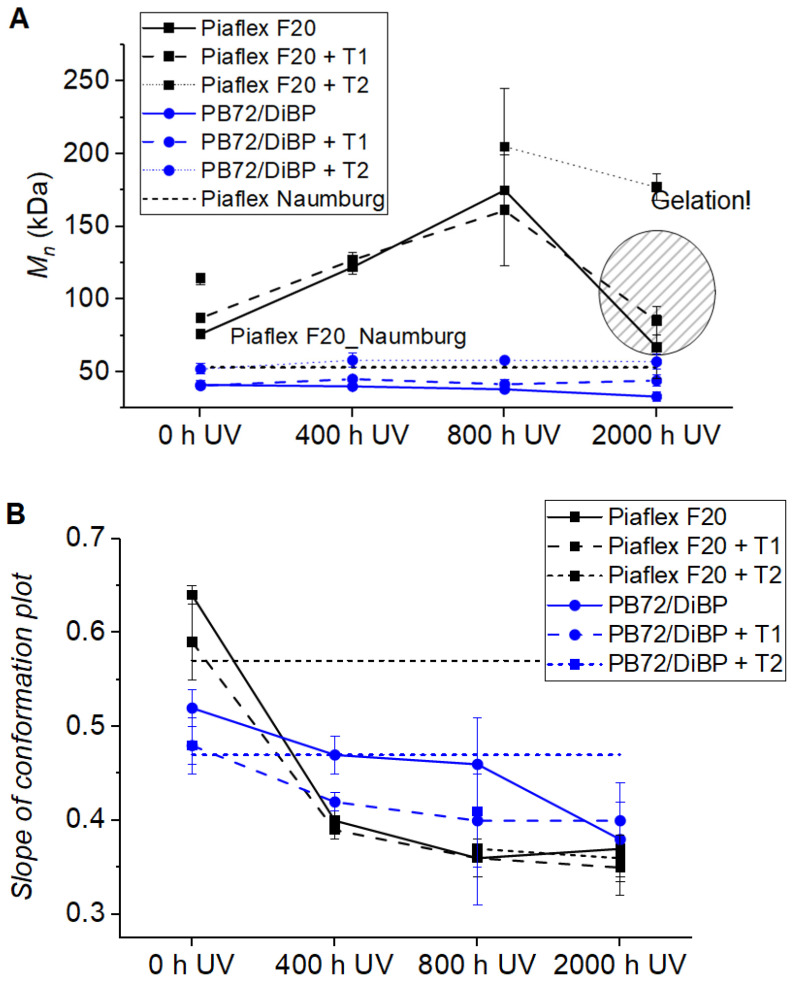
(**A**) Molar mass averages (SEC) of Piaflex F20 and Paraloid B72/DiBP during UV aging. At 53 kDa the value for Piaflex F20 from the Naumburg Cathedral (dashed line) is consistently lower than the artificially aged samples. (**B**) Slope of the conformation plots of Piaflex F20 and Paraloid B72/DiBP during UV aging. The dashed lines indicate values of real samples from the Naumburg Cathedral (black line: samples 38 and 74, blue line: samples 51, 56, 90).

In this study, FTIR spectroscopy and Raman spectroscopy showed that the polymers appeared to survive the artificial aging largely chemically unchanged. Only soluble acrylics can be investigated by SEC. At this point, results on Piaflex F20 are to be reported since data are available from both the naturally aged sample and the model substance after artificial aging under UV (Figure 12A). Both the molar masses and the architecture of the polymer molecules changed during artificial aging, with UV aging having the greatest influence. A clear increase in molar masses and a broadening of the distribution can be seen. The sample after 2000 h of UV irradiation is about 60% insoluble, i.e., cross-linked. Therefore, a decrease in the mean molar masses can be seen here since only the soluble fraction is recorded. The molar masses after the additional temperature aging give practically identical values.

The conformation plots show a clear increase in branching for Piaflex F20 already in the first 400 h of UV aging (slopes starting with c. 0.65) and further up to 2000 h (slopes of 0.35–0.40), which is attributed to the butyl moiety in the side chain (Figure 12B). In addition to significantly larger and branched molecules, shorter chain fragments are also formed, as shown in the thermal analysis (TGA) (see below). As Piaflex F20 carries a butyl moiety in the polymer chain, it is susceptible to crosslinking reactions and may become more difficult to solubilize on the glass windows with further aging. Thus, the re-dissolving of adhesives or films made of Piaflex F20 is likely to become problematic. However, the evaluation of measurements of naturally aged Piaflex samples from the Naumburg Cathedral (samples 38 and 74) shows conformations in the range of the merely thermally aged polymer (slopes of approx. 0.58) so that the real UV exposure in the cathedral must be assumed to be significantly lower than in the artificial weathering experiments carried out here.

In addition, SEC was performed for the mixture Paraloid B72/DIBP only after UV aging. With increasing irradiation time, the main molecular mass peak of Paraloid B72 shifts slightly to higher elution times, i.e., lower molecular masses, indicating chain scission processes. The molar mass averages decrease from approx. 40 kDa of the unaged Paraloid B72 to approx. 33 kDa after 2000 h of UV aging. No evidence of crosslinking due to initially increasing molecular mass, such as in Piaflex F20, was observed. This is consistent with findings in the literature, which attribute a higher tendency to crosslinking for acrylates with longer alkyl residues in the side group, such as Piaflex F20 (co-p-MMA/BMA), whereas acrylates with shorter side chains, such as Paraloid B72 (co-p-MME/EMA), are more prone to chain scission. For all acrylic resins, it was found that after aging under UV with a wavelength above 300 nm, the molecular weight distribution changed due to chain scissions on the one hand and coupling of macroradicals on the other hand. These reactions compensate for each other, maintaining the solubility of the polymers.

#### 3.2.3. Thermal Behavior

**Figure 13:**
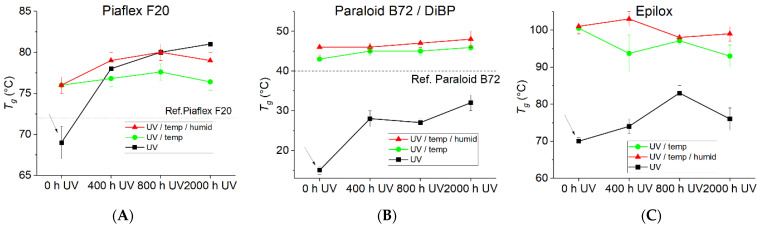
Glass transition temperatures of (**A**) Piaflex F20, (**B**) Paraloid B72/DiBP and (**C**) Epilox during artificial aging, together with reference values of materials from the Naumburg Cathedral. The arrows indicate unaged conditions.

**Figure 14:**
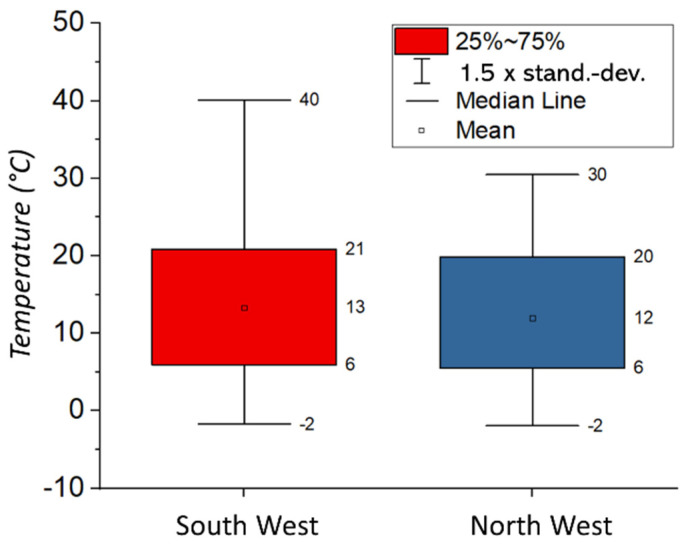
Measured surface temperatures on the stained glass windows in the Naumburg Cathedral, November 2016–October 2017 (data from [40]).

In its initial state, Piaflex F20 applied as a solution in toluene exhibits a lower T_g_ than the pure reference product, which is attributed to the solvent content (Table 1). Thermal aging (0 h UV) results in a significant increase in T_g_ to approx. 76 °C (Figure 13A). During both UV and combined aging (UV/temp), the T_g_ increases to values up to 9 K above the reference value, indicating embrittlement at lower temperatures due to aging. The effect of the additional increased humidity compared to the effect of temperature is negligible. The T_g_ of the historical Piaflex F20 specimens is 65–72 °C, which is lower than the values of the artificially aged specimens, indicating a less aged condition. However, the Piaflex F20 actually used on the Naumburg Cathedral is clearly more advantageous with respect to thermal stability, compared to Paraloid B72 (T_g_ = 38–40 °C, Table 1), especially with regard to the maximum surface temperatures of the glass windows of approx. 40 °C, as proven in the measurements (Figure 14) [40].

The TGA indicates an increase in heterogeneity of molecular mass distribution in the polymer, which was discussed already in the section about SEC. The TGA curve of the main degradation stage of Piaflex F20 becomes significantly broader with increasing UV aging and rises to higher temperature values (from c. 350 to c. 370 °C) (Figure S7). Increasing UV irradiation time is associated with an increase in contained low molecular weight components (mass loss to the beginning of the main degradation stage) in the artificially aged Piaflex F20, whereas the thermal aging programs each reduce their content (Figure S7). On closer inspection, a direct correlation between T_g_ and low molecular weight components is unlikely here. This behavior indicates that the changes in T_g_ are due more to changes in polymer structure than to the evaporation of the residual solvent.

The Paraloid B72 sample without plasticizer shows an unaged T_g_ significantly below the reference value, which increases to the final value of T_g_ = c. 46 °C during UV aging (Figure S8). Here, too, evaporation of the toluene can be assumed at first. The same value is already reached after thermal aging, while the previous UV aging no longer has any influence. The selected aging temperature of 80 °C (cumulatively approx. 1000 h) causes aging in both the pure and plasticized Paraloid B72, which has the effect of increasing the T_g_ by up to 10 K compared to the reference product. Pure UV aging leads to this value only after 2000 h (Figure 14). According to [9,42], the advantages of the acrylic resin Paraloid B72 are, among others, reversibility and high stability over time. Disadvantages of this polymer include limitations in use and storage in environments with high relative humidity and temperatures above 40 °C. This is probably due to the low glass transition temperature of Paraloid B72 (38–40 °C).

The PMA/DiBP sample initially shows an extremely low T_g_ = −14 °C, but this rises greatly to up to approx. 52 °C due to both UV and thermal aging (Figure 13B). Here, the experimentally determined reference value of the unaged PMA (without solvent) with T_g_ = 13 °C is clearly exceeded in both aging programs (Table 1). Thus, the PMA-DiBP mixture corresponding to the historical samples shows the same behavior as the plasticized model substance of Paraloid B72 (Figure S8). The T_g_ values achieved by artificial aging are also significantly higher than the values of the historical samples (T_g_ = 16–20 °C). This shows that artificial aging is significantly more advanced than natural aging. Accordingly, DiBP plasticizer could still be detected in the historical samples by PY-GC-MS.

During all thermal aging steps (without and with increased humidity, without and with UV irradiation), the Epilox shows a strong increase in T_g_ from 70 °C to approx. 93–103 °C (Figure 13C). The influence of humidity and UV radiation is negligible here. Under pure UV irradiation, the T_g_ initially rises moderately to 82 °C but drops again somewhat after 2000 h. Thus, epoxy resin shows similar behavior in artificial aging as the plasticized acrylate (Paraloid B72/DiBP). The content of low molecular weight components (as measured with TGA) increases slightly with UV irradiation and decreases somewhat with thermal aging (without and with increased humidity). Qualitatively, a decrease in low molecular weight components could partially explain the increase in T_g_. However, a post-crosslinking of the epoxy resin at elevated temperatures is more likely.

#### 3.2.4. Adhesive Strength

**Figure 15:**
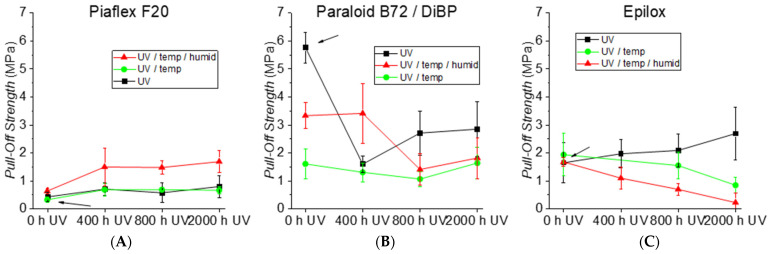
Pull-off strengths of (**A**) Piaflex F20, (**B**) Paraloid B72/DiBP and (**C**) Epilox after different stages of artificial aging. The arrows indicate unaged conditions.

For Piaflex F20 (Figure 15A), the adhesion values are generally very low, with usually the entire film being detached. Both pure UV aging and aging with UV and elevated temperature have hardly any influence on the film adhesion (σ = 0.3–0.7 MPa). After aging with UV and subsequent temperature and humidity exposure, significantly higher values were obtained (σ = 1.5–1.7 MPa). The reason could possibly be the incorporation of water with a slight plasticizing effect.

For Paraloid B72/DiBP (Figure 15B), significantly higher film adhesion is measured first (σ = c. 5.8 MPa) than for Piaflex F20. The high plasticizer content and the lower T_g_ of the polymer apparently allow the film to conform better to the surface of the glass. The UV and thermal aging programs clearly reduce the film adhesion to about half. Thermal aging without or with UV exposure has the strongest effect (σ = c. 1.1–1.7 MPa). The reason for this is the decrease in plasticizer content due to artificial aging. After the second aging program, the film adhesion of pure Paraloid B72 was measured. As expected, the values of pure Paraloid B72 were lower than those of the polymer with a plasticizer. However, the effect decreases with the loss of plasticizer, so that after thermal aging, both samples show practically the same values.

The films made of PMA/DiBP predominantly did not survive the second aging series (UV, without and with elevated temperature) as a continuous film. However, the values without aging (σ = 1.2 ± 0.2 MPa), after 400 h UV aging (σ = 1.7 ± 0.7 MPa) and after 2000 h (σ = 1.3 ± 1.0 MPa) showed a comparatively low film adhesion such as Piaflex F20 and thus a large difference to the plasticized Paraloid B72. The effect of DiBP as a plasticizer could be investigated by comparing Paraloid B72/DiBP and the non-plasticized Paraloid B72 (Figure S9). As the results of both experiments are comparable, the plasticizer does not seem to have a strong effect on the adhesion strength of the applied polymer films.

The results for the film adhesion of the acrylates (Piaflex F20, Paraloid B72/DiBP, Paraloid B72) show that there is a strong correlation to the T_g_ or the flexibility of the material. The “softer” a material is (lower T_g_), the higher the film adhesion. Environmental influences such as UV irradiation and temperature fluctuations have an effect on the development of coating adhesion in artificial aging. Here, however, there are no clear trends as with the T_g_. High humidity also has an influence on the film adhesion, in the case of acrylic resins, positively, and in the case of Epilox, negatively (Figure 15C).

To reproduce the character of the corroded and rough glass in the cathedral at least to some extent, Piaflex F20 and Epilox were additionally aged on sandblasted glass (Figure S9). Although Piaflex F20 initially exhibited slightly higher adhesion values on the roughened glasses, the effect of the surface after UV irradiation or thermal aging was negligible. The adhesion of the Epilox on the roughened glass was lower than on the smooth glass after UV aging. However, after UV aging without and with thermal exposure, it was greater than on the smooth glass.

## 4. Conclusions

Application-oriented publications [9,42] mention that an epoxy resin widely used today for bonding glass showed the worst performance in aging tests and yellowed significantly in a relatively short time. This phenomenon was also observed here for the (pre-aged) epoxy resin, Epilox. Whereas Paraloid B72, as an important protective agent, had been tested before for stability (for example, [9,21,29]), results from artificial aging for the polymers from production in the former German Democratic Republic (Piaflex F20, Epilox) as well as for PMA, including the mixture with plasticizer, seem not having been determined yet. The high aging stability of Paraloid B72 was confirmed in this study as well as in other publications [9,21,29]. Following from the relatively high glass transition temperature of Piaflex F20, for the future conservation of the glass windows already treated, it can be recommended to preserve the bondings and consolidations with Piaflex F20 as far as possible.

The color measurements showed that artificial aging leads to yellowing in Epilox and PMA. In particular, elevated temperatures and humidity are relevant influencing parameters. In comparison, Piaflex F20 yellows less and Paraloid B72 only slightly. On the other hand, plasticized Paraloid B72 yellows strongly under artificial temperature aging, which can be attributed to the plasticizer content.

The natural aging of Piaflex F20 resulted in comparatively small changes in molecular masses and conformation (Figure 12). However, both molar masses and architecture change significantly during artificial aging, with UV aging having the greatest influence. The results from SEC clearly show that Paraloid B72 mixed with DiBP is the more stable acrylate as compared to Piaflex F20.

The glass transition temperatures T_g_ of the acrylic polymers Piaflex F20 and PMMA, which are mainly used for the conservation of the glass windows, are—even in the naturally aged state—significantly above the surface temperatures on the stained glass windows in the Naumburg Cathedral (Figure 14) and thus seem to be well suited. Thus, it can be recommended to preserve the bondings and consolidations with these acrylics as far as possible. In contrast, the T_g_ of the PMA used as a coating, especially the mixture with plasticizer used as a lamination adhesive, are in the range of the most frequent temperatures in the cathedral. As the T_g_ is regularly and frequently exceeded, this probably has led to the observed flow but also to the inclusion of inorganic components (dust) in the polymer (Figure 2 and Figure 3). After artificial aging, the studied samples (Piaflex F20, PMA/DiBP, Paraloid B72, Paraloid B72/DiBP, Epilox) all show an increase in T_g_. While both UV irradiation and elevated temperatures have an influence on this, humidity plays a negligible role.

From the results of artificial aging applied in this study, only a few general trends could be derived. UV irradiation and extreme temperatures tend to show a stronger influence than humidity. A comparison of the naturally aged polymers from the cathedral with the artificially aged specimens shows that the naturally aged ones were less severely degraded. This speaks for the effectiveness of conservation and protection measures already implemented (e.g., exterior protective glazing since the 1970s) and against the very harsh conditions during the artificial aging performed. This study could gain only partial results on the adhesion on glass due to faulty experimental test set-up. Future studies should address this issue as well as the solubility and reversibility of the polymers, as presented in [9].

A trial restoration of nine stained glass panels from the west choir of Naumburg Cathedral revealed that it is not possible to remove the numerous historical restoration materials without risk and is not desirable for aesthetic reasons [43]. Due to the fundamental sensitivity of the historical conservation materials to UV, as demonstrated in this study, it was therefore decided to improve the climate in the area of the historical glass paintings by means of modern, internally ventilated protective glazing. The use of UV protective glasses for the protection not only of historical glass paintings but also for the minimization of further damage caused by old restorations represents a novelty in the preventive conservation of medieval glass paintings [43].

## Figures and Tables

**Table 1:** Measured glass transition temperatures Tg (°C) of historical samples, model polymers before and after artificial aging, and literature values [41]. Temp = thermal aging, Temp/Humid = thermal aging with elevated humidity.

Polymer	Historical Samples		Reference Material	Literature	Artificial Aging					
	1st Heat	2nd Heat			Before	Temp	Temp/Humid	UV (2000 h)	UV (2000 h)/Temp	UV (2000 h)/Temp/Humid
Piaflex F20	65–67	72–75	72	-	68	76	76	82	79	76
PMMA	63–65	80–84	-	105	-	-	-	-	-	-
PMA	16	20	13	6	-	-	-	-	-	-
PMA/DiBP	7–10	14–15	-		−14	16	-	30	31	-
Paraloid B72/DiBP	-	-	-	-	10	43	46	32	48	46
Paraloid B72	-	-	38	40	23	46	-	47	46	-
Epilox	-	-	-		70	100	100	71	89	93

## Data Availability

The data that support the findings of this study are available on request from the corresponding author.

## Supplementary Materials

The following supporting information can be downloaded at: https://www.mdpi.com/article/10.3390/polym15122595/s1, Figure S1. Raman spectra of Piaflex F20 after different steps of artificial ageing; Figure S2. Raman spectra of Paraloid B72/DiBP after different steps of artificial ageing; Figure S3. Raman spectra of Epilox after different steps of artificial ageing; Figure S4. Raman spectra of Paraloid B72 (without DiBP) after different steps of artificial ageing; Figure S5. FTIR Spectra of Epilox after UV irradiation, indicating post-crosslinking by decrease in intensity of free epoxy groups; Figure S6. FTIR Spectra of Epilox after UV and thermal ageing, indicating post-crosslinking by decrease in intensity of free epoxy groups; Figure S7. TGA curves of Piaflex F20 after different times of UV ageing; Figure S8. Tg values of PMA/DiBP and Paraloid B72 after UV and thermal ageing; Figure S9. Pull-Off Strengths of Piaflex F20 and Epilox on untreated and sandblasted (i.e., rough) glass.
