# Supplementary material for: Evaluation of the Composition, Thermal and Mechanical Behavior, and Color Changes of Artificially and Naturally Aged Polymers for the Conservation of Stained Glass Windows
Source: Polymers (Basel). 2023 Jun 7;15(12):2595. doi: 10.3390/polym15122595 (PMC10301440; doi:10.3390/polym15122595)
Supplement: Supplementary file 1 [file polymers-15-02595-s001.zip › polymers-2352545-supplementary.pdf]

Supporting Information

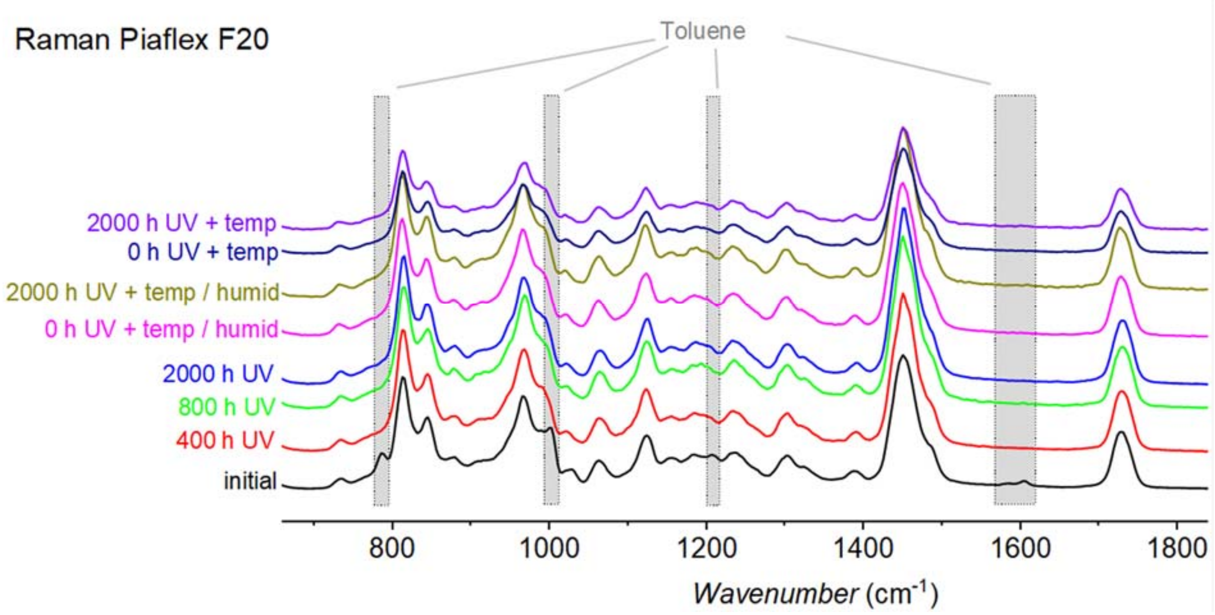

Figure S1: Raman spectra of Piaflex F20 after different steps of artificial ageing.

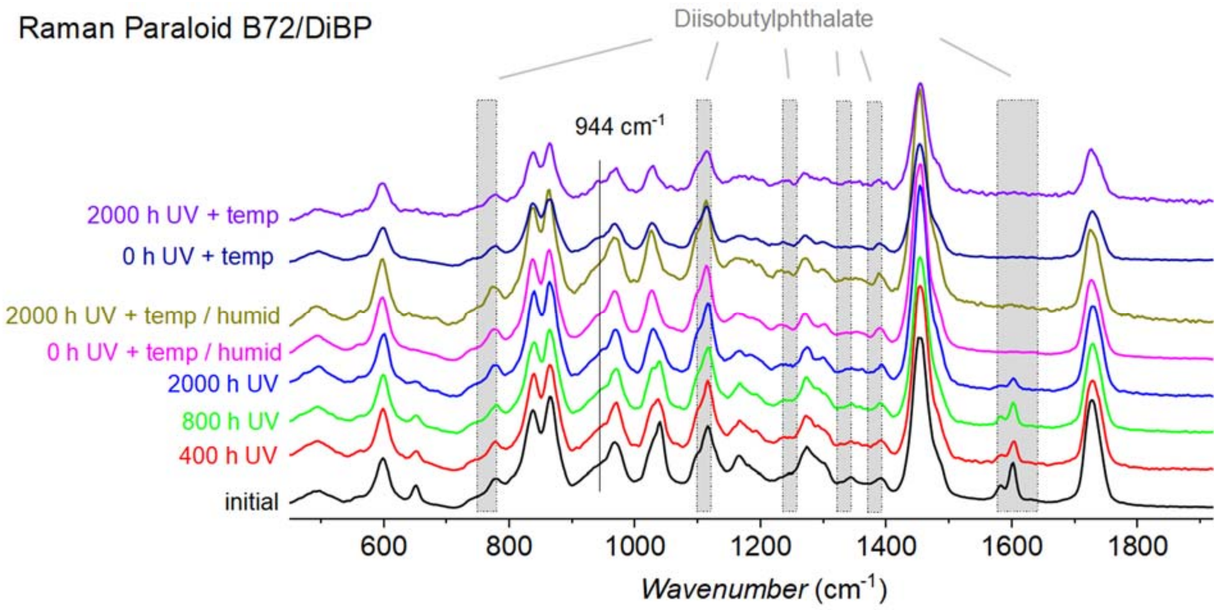

Figure S2: Raman spectra of Paraloid B72 / DiBP after different steps of artificial ageing.

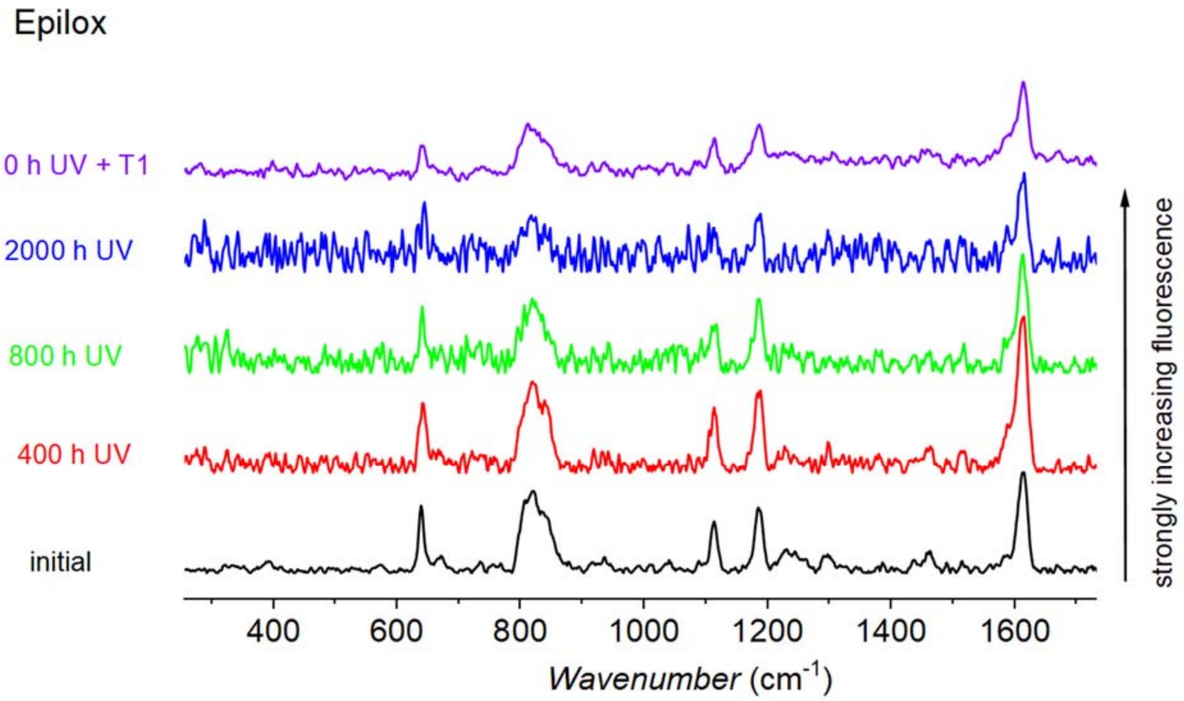

Figure S3: Raman spectra of Epilox after different steps of artificial ageing.

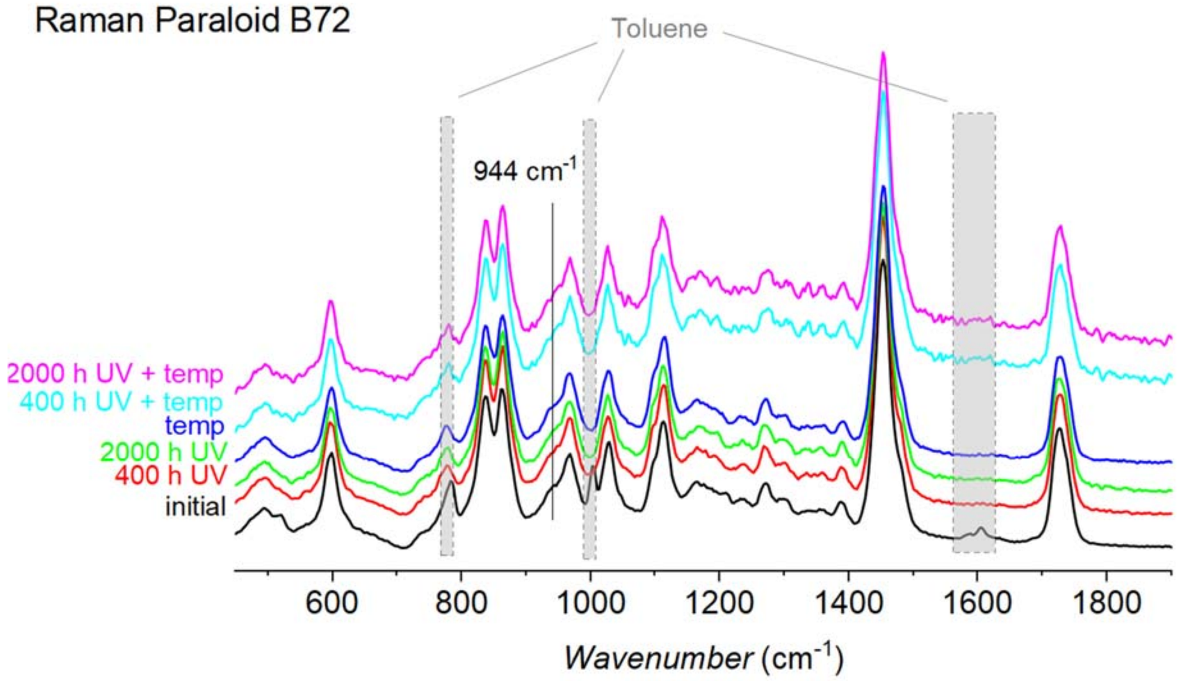

Figure S4: Raman spectra of Paraloid B72 (without DiBP) after different steps of artificial ageing.

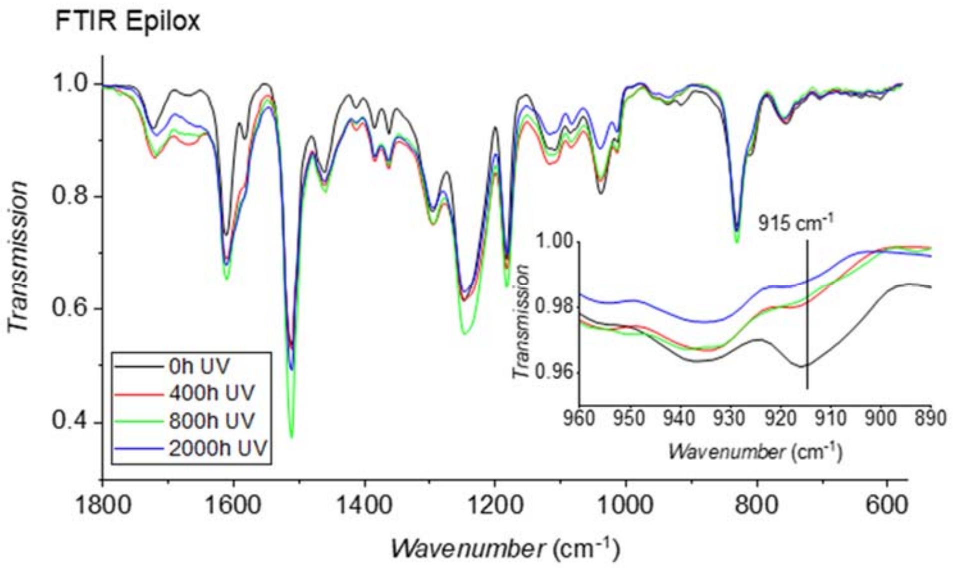

Figure S5: FTIR Spectra of Epilox after UV irradiation, indicating post-crosslinking by decrease in intensity of free epoxy groups.

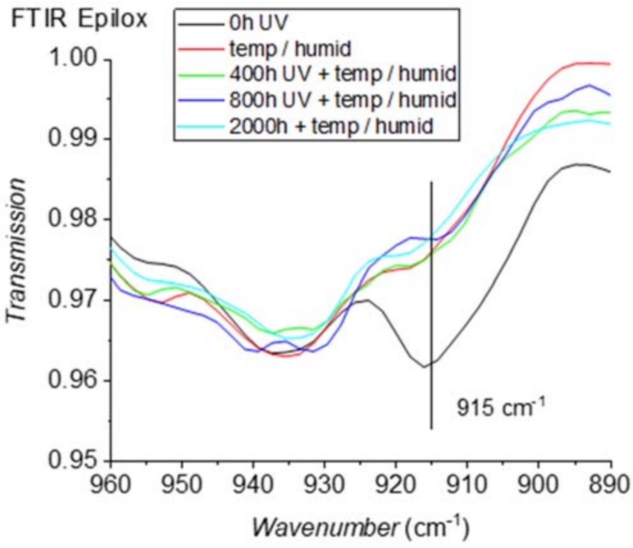

Figure S6: FTIR Spectra of Epilox after UV and thermal ageing, indicating post-crosslinking by decrease in intensity of free epoxy groups.

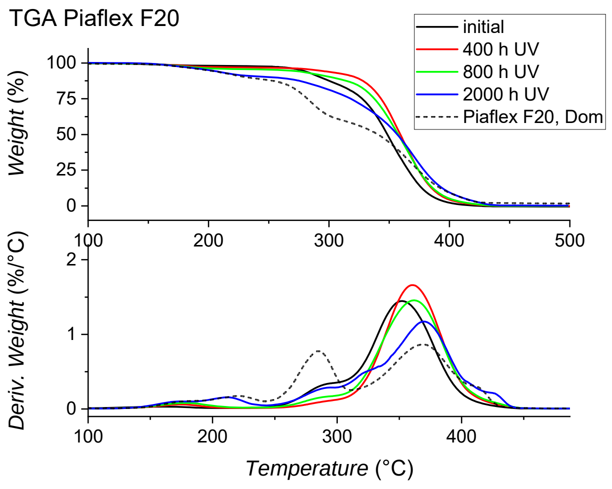

Figure S7: TGA curves of Piaflex F20 after different times of UV ageing.

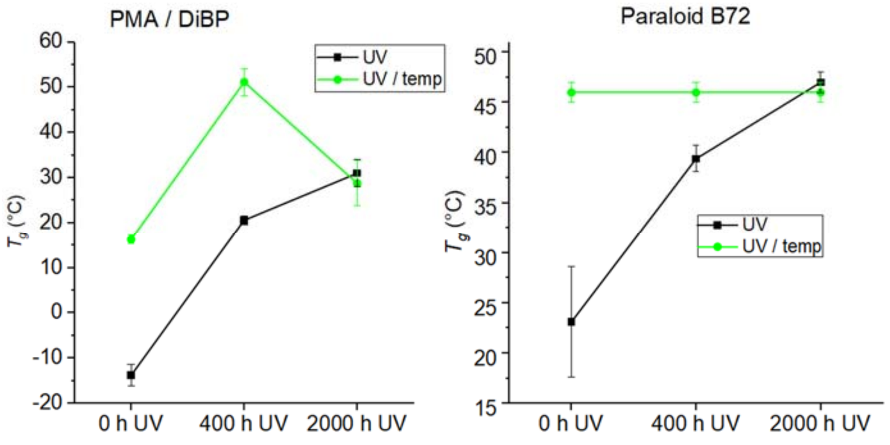

Figure S8: Tg values of PMA/DiBP and Paraloid B72 after UV and thermal ageing.

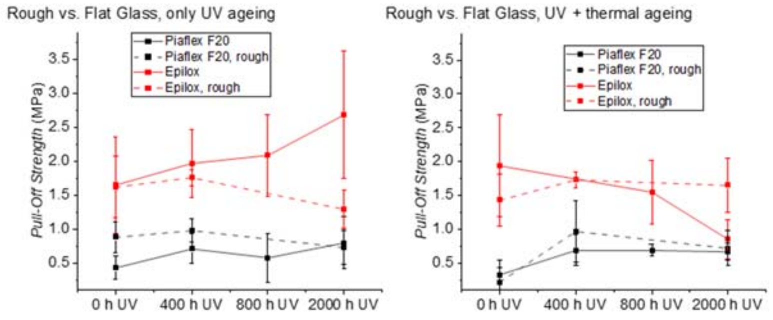

Figure S9: Pull-Off Strengths of Piaflex F20 and Epilox on untreated and sandblasted (i.e., rough) glass.
